# Changes in Intraocular Straylight and Visual Acuity with Age in Cataracts of Different Morphologies

**DOI:** 10.1155/2017/5649532

**Published:** 2017-07-31

**Authors:** Sonia Gholami, Nicolaas J. Reus, Thomas J. T. P. van den Berg

**Affiliations:** ^1^Rotterdam Ophthalmic Institute, Rotterdam, Netherlands; ^2^Amphia Hospital, Breda, Netherlands; ^3^Netherlands Institute for Neuroscience and Royal Netherlands Academy of Arts and Sciences, Amsterdam, Netherlands

## Abstract

**Purpose:**

To investigate the significance of difference in straylight of cataract eyes with different morphologies, as a function of age and visual acuity.

**Methods:**

A literature review to collect relevant papers on straylight, age, and visual acuity of three common cataract morphologies leads to including five eligible papers for the analysis. The effect of morphology was incorporated to categorize straylight dependency on the two variables. We also determined the amount of progression in a cataract group using a control group.

**Results:**

The mean straylight was 1.22 log units ± 0.20 (SD) in nuclear (592 eyes), 1.26 log units ± 0.23 in cortical (776 eyes), and 1.48 log units ± 0.34 in posterior subcapsular (75 eyes) groups. The slope of straylight-age relationship was 0.009 (*R* ^2^ = 0.20) in nuclear, 0.012 (*R* ^2^ = 0.22) in cortical, and 0.014 (*R* ^2^ = 0.11) in posterior subcapsular groups. The slope of straylight-visual acuity relationship was 0.62 (*R* ^2^ = 0.25) in nuclear, 0.33 (*R* ^2^ = 0.13) in cortical, and 1.03 (*R* ^2^ = 0.34) in posterior subcapsular groups.

**Conclusion:**

Considering morphology of cataract provides a better insight in assessing visual functions of cataract eyes, in posterior subcapsular cataract, particularly, in spite of notable elevated straylight, visual acuity might not manifest severe loss.

## 1. Introduction

The eye is an optical system with imperfections. Entering this optical system, light is refracted by the ocular media (e.g., cornea and crystalline lens) to form an image on the retina. However, part of this light is scattered by optical imperfections. Depending on the direction of scattering (forward or backward), it can have different influences on vision. The forward light scattering causes (intraocular) straylight or disability glare [1]. It produces undesired veiling of the retinal image which leads to reduced vision, glare, and other visual impairments. In young healthy eyes, almost 10% of the inbound light is scattered [2]. However, in eyes older than 50 years of age, this number increases considerably [3]. A phakic norm curve has been established that can be used as a reference for clinical practice [3]. Some pathological conditions, in particular cataract, increase the amount of intraocular straylight above normal. In clinical practice, a patient's visual complaints, ophthalmic examination with a slit-lamp, and measurement of visual acuity are the predominant scales for managing cataract. It should be noted that a slit-lamp examination provides only backscatter-based assessments. As the correlation between forward and backward light scattering has been shown to be small, methods that measure the amount of backscatter, such as slit-lamp examination, cannot reliably quantify straylight and glare [4–7]. Various studies have shown that straylight is a vision impairment that is not directly related to visual acuity and is only weakly correlated to it [3, 8]. A computerized purpose-built device, called C-Quant (Oculus Optikgeräte GmbH), measures the amount of ocular straylight and renders a parameter in the logarithmic unit (log(s)) with good reliability and repeatability [9–11].

As mentioned earlier, visual acuity is an important criterion in the cataract surgical decision-making process. However, various studies [12, 13] have shown that in a significant number of cataract cases, visual acuity is not an adequate measure to judge visual performance. Subsequent studies have supported this notion [14, 15]. Moreover, there have been reports of no change or even an increase in straylight after cataract surgery when the decision was made solely based on visual acuity [16]. The reason for this is that visual acuity only evaluates the impact of narrow-angle light spreading due to refractive errors and therefore can only measure a limited part of a patient's vision [13, 17]. Elliot et al. [7] expressed that additional visual tests were needed that could mirror visual loss but at the same time should be unrelated to visual acuity. They acknowledged the direct compensation method to quantify straylight as a standard technique to evaluate the validity of disability glare tests.

Recently, a literature review [18] established a norm curve for pseudophakic eyes and also a reference curve to estimate the amount of straylight to be expected after cataract surgery by introducing *straylight improvement* as a function of age and preoperative straylight. Although this reference is a good measure for cataract management in an average eye, it may overlook the influence of the type, location, and intensity of the cataract on the outcome because the type of cataract was not specified in the norm curve. To establish morphologically categorized references, we need a phakic norm stratified to the type of cataract. In the present study, we performed a literature review to identify relevant papers on straylight, age, and visual acuity in three common types of cataract. In addition, we recalculated the significance of the relation between straylight and visual acuity with taking cataract morphology into account. The published studies included in this literature review were evidenced individually that such correlation varies from one type of cataract to another. The population sizes and severities of the cataracts were different across these studies. We consider the relatively large final number of observations and their diverse degrees of cataract intensity as the strength of this study to improve the generalizability of the results.

## 2. Materials and Methods

This study includes two parts. The first part encompasses a comprehensive literature review to study the effect of different cataract morphologies on straylight and to determine models for straylight values as a function of age for different types of cataract. Second, we calculated the correlations between straylight and visual acuity, the amount of progression of straylight and visual acuity from those of a normal group, and the ratios of straylight to age and visual acuity in each cataract group.

A literature examination was carried out including all available studies that reported straylight values, measured with a C-Quant instrument (Oculus Optikgeräte GmbH), in cataract eyes with specification of its morphology. The language of the articles and age, gender, and race of the participants had no influence in this process. All papers provided information on intraocular straylight, age, and visual acuity of participants with the specification of the type of cataract. All papers had excluded patients with a history of ocular surgery or diseases, diabetic retinopathy, glaucoma, and age-related macular degeneration. We considered data with expected standard deviation (ESD) of 0.12 log units or less reliable for analysis.

PubMed, Medline, and Google Scholar were the scientific databases we screened using the following keywords: *straylight, C-Quant, age, visual acuity, cataract, cataract morphology, cataract classification, LOCS III, nuclear cataract, cortical cataract and posterior subcapsular cataract (PSC)*. In case of overlapping data in the studies, the one with the larger population was included for the review. Five papers met the eligibility criteria: de Waard et al., Nischler et al., Bal et al., Congdon et al., and Filgueira et al. [6, 19–22]. Because of lack of the desired data in four cases, we contacted the corresponding authors. In one case, there was no response; therefore GSYS2.4 (a graph digitizing system developed by Nuclear Reaction Data Center, University of Hokkaido, Japan) was used to extract data from the published graphs.  Table 1 shows which data were reported by the five included studies. It has to be noted that the various studies classified the types of cataract differently based on LOCS (Table 2).

Data from all five articles were used to develop the log(s)-age normative curves for the three types of cataract. The correlations between the two variables were calculated and compared with each other. We calculated the normally expected mean straylight value for each cataract type, all types of cataract combined and the control group by using the log(s)-age normative equation obtained by van den Berg et al. [23], which reads

(1) $$\begin{array}{c} \log\;\left(straylight\;parameter\right)=\log\left(s\right)=0.9+\log\;\left(1+{\left(age/65\right)}^{4}\right). \end{array}$$

The results were compared with the measured straylight values. The residuals are displayed by using histograms. To study the possible differentiative impact of morphology on the progressive process of cataract, we used the largest control group—which belonged to Nischler et al.—with the best straylight and visual acuity values. We then compared the straylight and visual acuity of each cataract group in each study by connecting the mean values to those of the control group using arrows to show the magnitude and direction of progression. The slopes and lengths of the arrows were compared with each other. The correlations between log(s) and logMAR visual acuity values were calculated and compared with each other. We also calculated the ratio of straylight to age and visual acuity for each type of cataract; the results are illustrated using box-and-whisker plots. The log(s)-logMAR normative curves for each type of cataract are also derived using data from all five articles.

Linear regression analysis was performed with Excel software (2010, Microsoft Corporation) and SPSS Statistics 21 (IBM Corporation) on the straylight values—log(s)—to describe it as a function of age and logMAR visual acuity. Unpaired *t*-tests were used to calculate the significance of differences in means (±95% CI) between each study and the normative curve of each cataract type. The significance level was set at *P* value less than 0.05.

## 3. Results

### 3.1. Comprehensive Review

**Figure 1:**
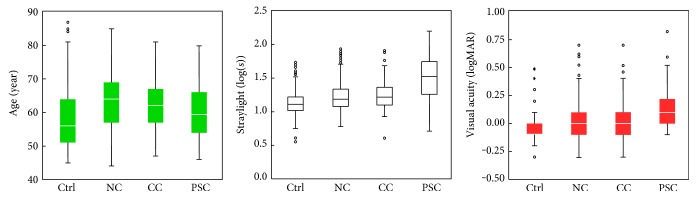
Age, intraocular straylight, and best-corrected visual acuity plotted for cataract and control groups. Straylight and visual acuity differed significantly from PSC to the other cataracts and control group. (NC: nuclear cataract; CC: cortical cataract; and PSC: posterior subcapsular cataract).

As explained in Materials and Methods, five reports fulfilled the eligibility criteria.  Table 3 shows a summary of outcomes of each study.  Table 4 shows the outcomes for each type of cataract, all types of cataract combined, and the control group.  Figure 1 illustrates age, visual acuity, and straylight distributions in each cataract group.

**Figure 2:**
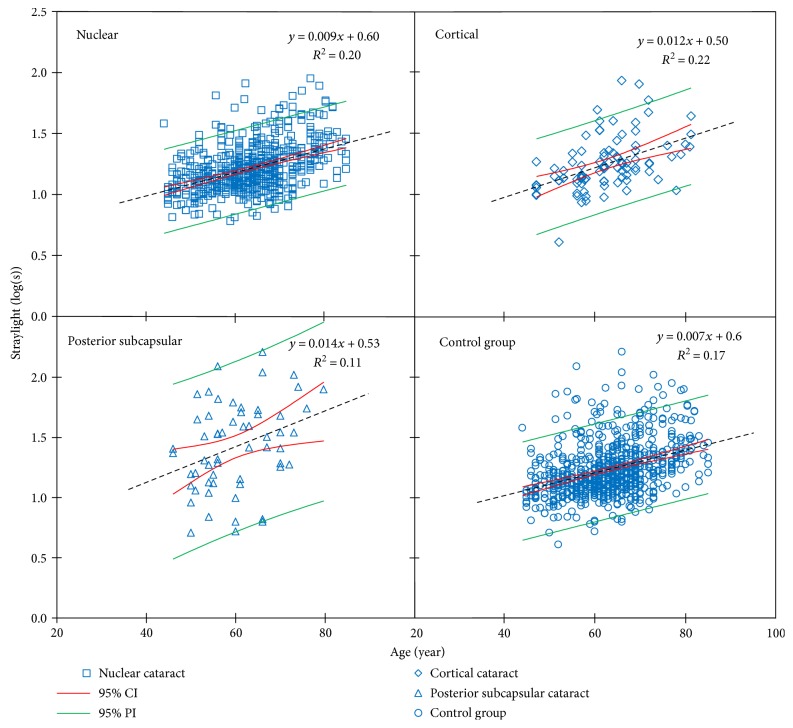
Linear models of log(s)-age dependency for nuclear cataract derived from four studies, cortical cataract derived from two studies, posterior subcapsular cataract derived from three studies and control group are plotted. Black dotted lines are the regression lines.

**Figure 3:**
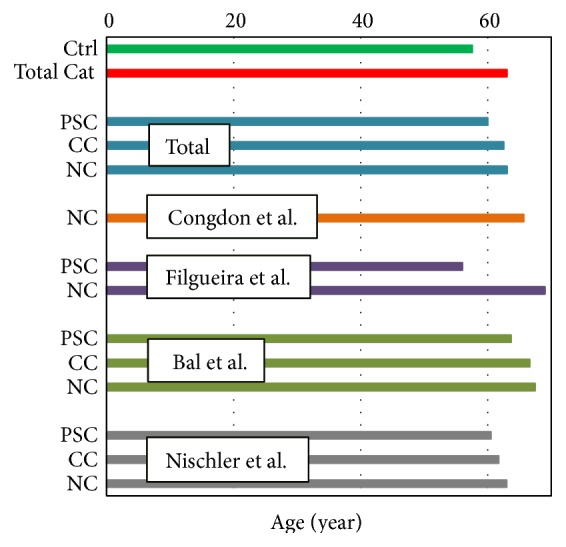
Mean age of each type of cataract in each published study. In average, patients with PSC cataract are the youngest. (Ctrl: control group; NC: nuclear cataract; Total cat: all cataract groups combined; CC: cortical cataract; and PSC: posterior subcapsular cataract).

The evaluations concerning log(s)-logMAR-related analyses were based on 776 total observations, with mean visual acuity of 0.02 ± 0.18 log units (range −0.30 to 0.70 log units) and mean straylight of 1.23 ± 0.22 log units (range 0.61 to 2.09 log units). The total number of cataract eyes was 725 for evaluations concerning log(s)-age-related analyses with a mean age of 63 ± 9 years (range 44 to 85 years of age). Figures 2 and 3 show the log(s)-age and log(s)-logMAR linear regressions for studies comprising the required data.

### 3.2. Effect of Cataract Morphology on Straylight

Straylight varied as a function of cataract morphology (Table 3); it was significantly higher in the three cataract groups (1.22 ± 0.20 log units in nuclear cataract, 1.26 ± 0.23 log units in cortical cataract, and 1.48 ± 0.34 log units in PSC) compared to the control group (1.12 ± 0.16 log units, *P* < 0.05). In addition, in all cataracts combined, straylight was significantly increased (1.26 ± 0.12 log units) relative to the control group (*P* < 0.05).

### 3.3. Correlation with Age

Straylight showed the highest correlation with age in Congdon et al.'s nuclear group (*R* ^2^ = 0.36, *P* < 0.05), and it showed no to a very weak correlation in several other groups (Table 3).  Figure 1 shows phakic normative curves for each type of cataract; the data were derived from 574 eyes of nuclear, 93 of cortical, and 58 eyes of PSC. Overall, cortical cataract showed the highest correlation between log(s) and age (*R* ^2^ = 0.22, *P* < 0.05), and the overall PSC showed the lowest correlation between the two variables (*R* ^2^ = 0.11, *P* < 0.05) (Table 4).  Figure 2 shows reference curves for cataracts and control group. The overall relationships read as

(2) $$\begin{array}{c} straylight\;value=0.009\;\times \;age\;+\;0.60\;\left(nuclear\;group;R^{2}=0.20,P<0.05\right),\\ straylight\;value=0.012\;\times \;age\;+\;0.50\;\left(cortical\;group;R^{2}=0.22,P<0.05\right),\\ straylight\;value=0.014\;\times \;age\;+\;0.53\;\left(PSC\;group;R^{2}=0.11,P<0.05\right), \end{array}$$

whereas that of the control group reads

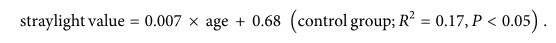
(3)

Mean straylight values are displayed in Tables 2 and 3. The figures show differences in different studies.

**Figure 4:**
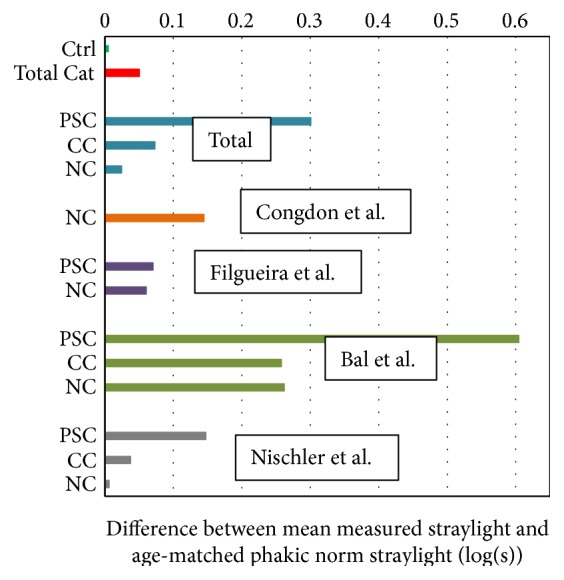
Differences between the mean straylight value in patients with different types of cataract for the various studies and the age-matched straylight value derived from the phakic norm curve by van den Berg et al. [23] are plotted. In all data, straylight in patients with PSC cataract showed the highest deviation from that of a noncataract (phakic) group. (Ctrl: control group; Total cat: all cataract groups combined; NC: nuclear cataract; CC: cortical cataract; and PSC: posterior subcapsular cataract).

The mean age of each group is depicted in Figure 3. Using the log(s)-age norm curve equation obtained by van den Berg et al. [23], we calculated the expected mean straylight for each cataract type of each study, overall cataract types, all cataract types combined, and control groups. The results were compared with the measured straylight values. The residuals are displayed in Figure 4. We must remind the reader that not every study provided information on age (Table 1). Among three cataract groups, the mean straylight of PSC group showed the highest difference from the expected mean straylight of an age-matched phakic group; by contrast, nuclear group showed the smallest difference. The same figure shows negligible difference between measured and expected straylight in control group.

### 3.4. Correlation with Visual Acuity

Straylight showed the highest correlation with logMAR visual acuity in de Waard et al.'s cortical group (*R* ^2^ = 0.29, *P* < 0.05) and the lowest correlation in Nischler et al.'s cortical group (*R* ^2^ = 0.00, *P* = 0.99). Overall, PSC showed the highest correlation between straylight and logMAR visual acuity (*R* ^2^ = 0.34, *P* < 0.05) and cortical cataract showed the lowest correlation, however significant, between the two variables (*R* ^2^ = 0.13, *P* < 0.05). The relations and correlation coefficients between log(s) and logMAR visual acuity are reported in Tables 3 and 4.

**Figure 5:**
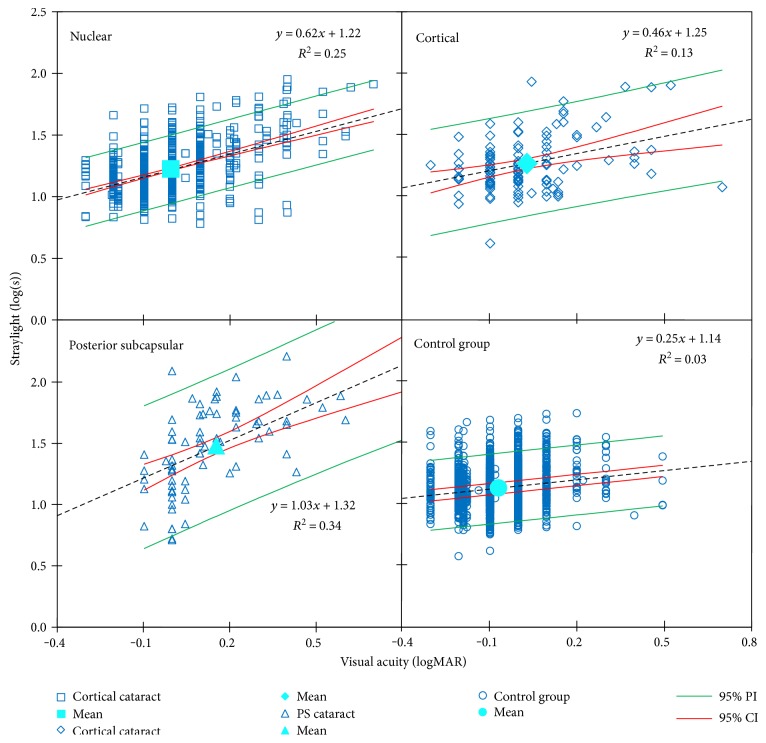
Linear models of log(s)-logMAR visual acuity dependency for nuclear cataract derived from five studies, cortical cataract derived from three studies, PSC cataract derived from four studies and control group are plotted. Black dotted lines are the regression lines.

Figure 5 shows log(s)-logMAR reference curves for cataracts and control group. The overall relationships read as

(4) $$\begin{array}{c} straylight\;value=0.62\;\times \;visual\;acuity\;+\;1.22\;\left(nuclear\;group;R^{2}=0.25,P<0.05\right),\\ straylight\;value=0.33\;\times \;visual\;acuity\;+\;1.24\;\left(cortical\;group;R^{2}=0.13,P<0.05\right),\\ straylight\;value=1.03\;\times \;visual\;acuity\;+\;1.34\;\left(PSC\;group;R^{2}=0.34,P<0.05\right), \end{array}$$

whereas the norm of the control group reads as

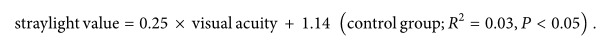
(5)

From the above relationships, one can see that straylight varies as a function of morphology. Patients with PSC for a similar logMAR visual acuity have a higher straylight than the other cataracts and control group.

### 3.5. Cataract: Progression from Healthy Eyes

**Figure 6:**
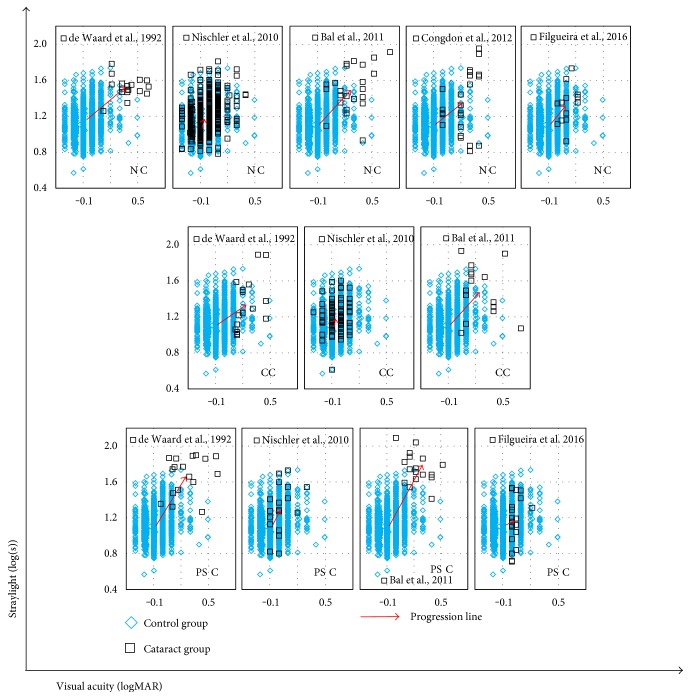
Progression of cataracts from control group is illustrated by arrows originating from mean straylight and mean visual acuity of the control group towards those of each type of cataract in each individual study. (NC: nuclear cataract; CC: cortical cataract; and PSC: posterior subcapsular cataract).

We estimated the amount of progression of mean straylight and mean visual acuity from those of the control group in each individual study and cataract groups. The progression lines are demonstrated in Figure 6. Data showed that PSC in Bal et al. had the highest progression from noncataract status in terms of both straylight (ΔSL = 0.68 log units) and visual acuity (ΔVA = 0.37 log units). However, with respect to the progression of individual variables, the mean visual acuity increased the most in de Waard et al.'s nuclear group (ΔVA = 0.46 log units), whereas its mean straylight value increased (ΔSL = 0.42 log units) less than that of Bal et al.'s PSC group. The mean visual acuity deteriorated the least in Nischler et al.'s nuclear and cortical groups.

### 3.6. Ratio between Straylight and Age and Visual Acuity

**Figure 7:**
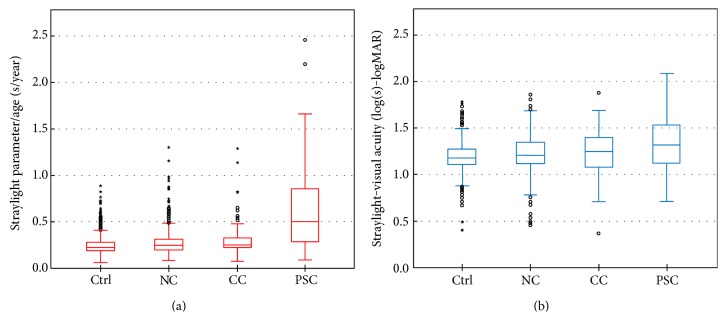
(a) Ratio between straylight parameter and age for each type of cataract: the median of such ratio is significantly higher in the PSC group. (b) The same is valid for straylight-visual acuity. Both cases suggest that straylight is highest in the patients with PSC group albeit lower and/or similar age and visual acuity than/as the other cataracts. (Ctrl: control group; NC: nuclear cataract; CC: cortical cataract; and PSC: posterior subcapsular cataract).

The ratios between straylight and age and between straylight and visual acuity are illustrated using box-and-whisker plots (Figure 7()). The median of straylight parameter (s)/age (year) had the lowest value in nuclear cataract group and the highest value in PSC group, albeit with a rather more skewed distribution comparing the two other cataract groups. The differences in medians of the PSC group and the other two cataract groups were statistically significant. The median of log(s)-logMAR showed similarly lower values in nuclear and cortical groups in comparison to that of PSC group.

## 4. Discussion

The application of the findings of this literature review is limited by the restricted range of the severity of cataracts and the difference in age between the studies. However, it is a good place to start studying the distinctive relationship between cataract morphology and visual functions. There is a strong correlation between cataract morphology, the intensity of lens opacification, and impairment of visual functions (i.e., straylight and visual acuity).

From the results, we found that PSC population was generally younger, which is in agreement with the literature [24–27] reporting that the average age of the population developing or undergoing surgery for PSC is younger than that for other types of cataract.

In the present study, the log(s)-age dependencies were obtained for cataracts of different morphologies. Among five published articles used in this literature review, four could be used for the nuclear, three for the PSC, and two for the cortical log(s)-age dependency equations (Table 1). These equations cannot be considered normative reference curves, because the different studies made a severity selection of the cataract populations. This must have influenced (weakened) the dependencies. The slopes of the dependency equations varied from one cataract group to another, but the differences were not statistically significant. The slope of the dependency function of the nuclear cataract was close to that of the control group. The reason is that Nischler et al.'s nuclear group with patients with rather good vision was remarkably larger than the rest. The differences between cataracts and control groups, as mentioned earlier, were small, with large overlap between the cataract and control populations. This points at limited validity of the LOCS cataract grading. Although LOCS serves to improve the grading and classifying slit-lamp observation, it is not precise for assessing function. As mentioned in the introduction, there is a weak relation between backscatter and forward scatter; therefore, a slit-lamp-based measurement cannot be a reliable means to quantify forward scatter. The correlation between log(s) and age also varied between cataract groups and control group; it was the highest in cortical cataract and the lowest in PSC.

In each cataract group, the difference in the mean straylight values of individual studies and the respective dependency function was significant. This can be explained by different levels of cataract severity and significant difference in the number of eyes of the largest study and the rest. Such difference was not observed between the slopes of each study and the respective dependency functions. It appeared that the mean straylight values of the reference curves were moderately closer to those of Nischler et al.'s nuclear and cortical groups. Nischler et al. covered the major part of the overall data in these two groups; therefore, it is no surprise that it leads the outcomes. The reason Nischler et al. had the lowest mean values in straylight and visual acuity may be due to the fact that the patients were active drivers; therefore, they had comparatively better vision than their age-equivalent peers in other studies. The lowest straylight belonged to Filgueira et al.'s PSC. This is a deviant behavior as PSC in every other study had the highest straylight value. This group's visual acuity was almost as low as that of Nischler et al. Recruiting patients with eyes at the early stage of cataract in these two studies can explain these results (Table 2). When we left out data from Nischler et al. and Filgueira et al., the difference in means of cataract groups became very small, whereas the mean straylight of PSC was approximately 0.3 log units higher than that of other cataracts. This is in agreement with the finding by Elliott et al. [28, 29] that in the advanced stages of cataracts, for patients with PSC, visual acuity alone is not an adequate assessment of visual performance and cataract management. The straylight curve established for normal phakic eyes [23] shows that straylight increases strongly with age with a logarithmic relation (to the power of 4). The change in straylight shows stable behavior in young eyes and considerably increases over 50 years of age. However, our findings showed rather linear relationship between log(s) and age. This may be related to the selection based on severity. We also found that the control group in Figure 4 shows the phakic reference norm works very well.

The correlation between log(s) and logMAR visual acuity varied from none to a moderate one in individual studies and within cataract types, but it never was strong. Overall, no type of cataract showed strong log(s)-logMAR correlation. In clinical practice, this means straylight cannot be predicted on the basis of visual acuity for any type of cataract. Figure 6 shows that, overall, in PSC group straylight deteriorated faster than visual acuity. Some studies [30, 31] found that with increasing the severity of posterior capsule opacification (PCO), visual acuity and straylight deteriorate, albeit with different rates; the PCO severity-log(s) relation is linear, whereas the PCO severity-logMAR is curvilinear [31]. Therefore, straylight is more sensitive to the changes in PCO severity than visual acuity. Kruijt and van den Berg [32] also discussed this difference for localized processes.

Regardless of severity of cataracts, the present study supports the notion that the straylight is the highest in PSC. Fluctuations in density and discontinuous refractive index can be responsible for such amplification [33, 34]. The difference between log(s)-logMAR dependency slopes of cataracts of different morphologies and their correlation coefficients is notable. The distinction between PSC and noncataract eyes is especially remarkable. The log(s)-logMAR progression of cataracts from a control group in our study also showed that PSC deteriorated the most in terms of visual functions. Therefore, it can be inferred that patients with this type of cataract would benefit the most from surgery. However, to draw definite conclusion, further studies on the improvement of visual functions after cataract surgery considering cataract morphologies are necessary. The results presented in Figure 4 show that the age-corrected mean straylight values of Bal et al. in every cataract groups are higher than those of other studies. Unlike patients recruited in the other studies, these patients were listed for cataract surgery. When Bal et al.'s patients were excluded from analysis, PSC had the worst visual acuity; the change in visual acuity in all cataract groups was in average 0.03 log units. The changes in straylight of nuclear and cortical groups were negligible, but it decreased about 0.11 log units in PSC. Therefore, the difference in mean straylight of PSC remained remarkably higher than the other cataracts and the correlation with age decreased (*R* ^2^ = 0.04, *P* = 0.22). The slopes of the new reference curves remained almost unchanged. We observed no change in the new log(s)-logMAR correlation in any cataract group, whereas the slopes changed, albeit insignificantly, with the average change of 0.06. Although the effect of excluding Bal et al.'s data on our analysis was unimportant, one needs to recognize the relatively small size of this study as an effective factor in this context.

It should be noted that correlations that were significant in one or some studies, and were not in the other(s), were in fact significant in the whole cataract group. However, this cannot be said about the whole data (different types of cataracts combined), because of different morphologies and eventually different optical dynamics.

## 5. Conclusion

We confirm that straylight in cataract eyes varies rather independently from age and best-corrected visual acuity. The independence of these two aspects of crystalline lens was speculated to be caused by different optical processes of remarkably different scales [3]. We found that, in accordance to the literature, to assess visual functions of cataracts, the analysis should consider cataract morphology. This becomes more crucial in PSC, where the general visual acuity might not show severe loss, but a remarkable increase of straylight above the cutoff value of 1.40 log units [35] can have negative effect on the quality of life. The norm curves obtained in this literature review serve to distinguish the particular effect of each type of cataract, from early-stage to mild, on visual impairment. However to generalize our results, scrutinize their validity in more severe cataracts, and to develop postoperative straylight improvement references, further studies are needed.

## Figures and Tables

**Table 1:** Red circles show deficient data, and green circles show available data in each individual study.

Cataract	Study (year)	Age	SL	VA
Nuclear	Filgueira et al. (2016)	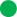	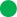	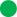
	Congdon et al. (2012)	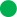	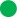	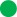
	Bal et al. (2011)	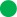	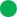	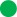
	Nischler et al. (2010)	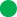	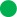	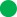
	de Waard et al. (1992)	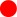	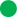	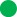
				
Cortical	Filgueira et al. (2016)	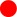	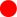	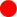
	Congdon et al. (2012)	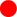	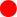	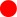
	Bal et al. (2011)	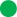	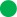	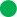
	Nischler et al. (2010)	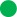	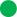	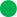
	de Waard et al. (1992)	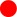	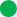	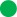
				
Posterior subcapsular	Filgueira et al. (2016)	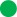	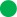	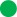
	Congdon et al. (2012)	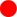	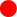	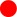
	Bal et al. (2011)	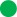	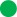	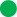
	Nischler et al. (2010)	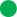	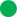	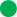
	de Waard et al. (1992)	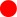	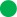	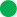

**Table 2:** Range of intensity defined for each type of cataract in the studies.

Study	Cataract definition
Filgueira et al. (2016)	Early age-related cataracts: nuclear (NO = 1 and 2), posterior subcapsular (*P* = 1 and 2)
Congdon et al. (2012)	Nuclear (NO ≥ 3)
Bal et al. (2011)	Nuclear (NO > 2, NC > 2, *C* ≤ 2, *P* ≤ 1), cortical (NO ≤ 2, NC ≤ 2, *C* > 2, *P* ≤ 1), posterior subcapsular (*C* ≤ 2, *P* > 1)
Nischler et al. (2010)	Nuclear (2 ≤ NO ≤ 4, 2 ≤ NC ≤ 4, *C* < 2, *P* ≤ 1.5), cortical (NO < 2, NC < 2, 2 ≤ *C* ≤ 4, *P* ≤ 1.5), posterior subcapsular (NO < 2, NC < 2, *C* < 2, 1.5 ≤ *P* ≤ 4)
de Waard et al. (1992)	Advanced age-related cataracts (morphologically not categorized)

**Table 3:** Overview of the analysis on the data derived from raw data or published plots of each individual study (NC: nuclear cataract; CC: cortical cataract; and PSC: posterior subcapsular cataract).

Cataract	First author (year)	Eyes (*n*)	Mean ± SD			log(s)-age		log(s)-logMAR	
			Age (Y)	VA (logMAR)	SL (log(s))	Dependency	*R* ^2^	Dependency	*R* ^2^
NC	Filgueira (2016)	14	69 ± 18	0.10 ± 0.08	1.33 ± 0.21	log(s) = 0.008 × age + 0.75	0.02	log(s) = 0.14 × logMAR − 0.08	0.14
	Congdon (2012)	24	65 ± 10	0.22 ± 0.14	1.36 ± 0.33	log(s) = 0.019 × age + 0.12	0.36	log(s) = 0.82 × logMAR + 1.17	0.13
	Bal (2011)	23	67 ± 9	0.28 ± 0.18	1.50 ± 0.24	log(s) = 0.001 × age + 1.54	0.00	log(s) = 0.51 × logMAR + 1.36	0.14
	Nischler (2010)	512	63 ± 9	−0.05 ± 0.12	1.19 ± 0.17	log(s) = 0.008 × age + 0.65	0.21	log(s) = 0.44 × logMAR + 1.21	0.10
	de Waard (1992)	18	NA	0.39 ± 0.14	1.54 ± 0.16	NA	NA	log(s) = 0.01 × logMAR + 1.42	0.08
									
CC	Filgueira (2016)	NA	NA	NA	NA	NA	NA	NA	NA
	Congdon (2012)	NA	NA	NA	NA	NA	NA	NA	NA
	Bal (2011)	15	67 ± 7	0.25 ± 0.20	1.48 ± 0.29	log(s) = 0.020 × age + 0.13	0.26	log(s) = −0.28 × logMAR + 1.55	0.04
	Nischler (2010)	78	62 ± 8	−0.06 ± 0.09	1.20 ± 0.17	log(s) = 0.008 × age + 0.69	0.17	log(s) = 0.13 × logMAR + 1.21	0.00
	de Waard (1992)	16	NA	0.24 ± 0.13	1.34 ± 0.29	NA	NA	log(s) = 1.22 × logMAR + 1.04	0.29
									
PSC	Filgueira (2016)	20	56 ± 5	0.03 ± 0.05	1.17 ± 0.27	log(s) = 0.013 × age + 0.42	0.06	log(s) = 0.04 × logMAR − 0.02	0.11
	Congdon (2012)	NA	NA	NA	NA	NA	NA	NA	NA
	Bal (2011)	20	64 ± 9	0.30 ± 0.22	1.79 ± 0.20	log(s) = 0.001 × age + 1.77	0.00	log(s) = 0.13 × logMAR + 1.76	0.02
	Nischler (2010)	18	61 ± 8	0.02 ± 0.11	1.30 ± 0.27	log(s) = 0.000 × age + 1.19	0.00	log(s) = 1.02 × logMAR + 1.28	0.17
	de Waard (1992)	17	NA	0.26 ± 0.18	1.67 ± 0.21	NA	NA	log(s) = 0.33 × logMAR + 1.58	0.08

**Table 4:** Overview of the analysis on collected data from individual studies for each cataract group.

Group	Number of eyes		Mean ± SD			log(s)-age		log(s)-logMAR	
	SL-age	SL-VA	Age (year)	VA (logMAR)	SL (log(s))	Dependency	*R* ^2^	Dependency	*R* ^2^
Nuclear	573	592	63 ± 9	−0.01 ± 0.16	1.22 ± 0.20	log(s) = 0.009 × age + 0.60	0.20	log(s) = 0.62 × logMAR + 1.22	0.25
Cortical	93	109	62 ± 8	0.03 ± 0.18	1.26 ± 0.23	log(s) = 0.012 × age + 0.50	0.22	log(s) = 0.33 × logMAR + 1.24	0.13
Posterior	58	75	60 ± 8	0.15 ± 0.20	1.48 ± 0.34	log(s) = 0.015 × age + 0.53	0.11	log(s) = 1.03 × logMAR + 1.32	0.34
All cataracts	724	776	63 ± 9	0.02 ± 0.18	1.23 ± 0.22	log(s) = 0.009 × age + 0.64	0.14	log(s) = 0.68 × logMAR + 1.24	0.26
Control	1761	1761	57 ± 8	−0.07 ± 0.11	1.12 ± 0.16	log(s) = 0.008 × age + 0.68	0.17	log(s) = 0.25 × logMAR + 1.14	0.03
